# Epidemiology of Inflammatory Bowel Diseases: A Population Study in a Healthcare District of North-West Italy

**DOI:** 10.3390/jcm12020641

**Published:** 2023-01-13

**Authors:** Gian Paolo Caviglia, Angela Garrone, Chiara Bertolino, Riccardo Vanni, Elisabetta Bretto, Anxhela Poshnjari, Elisa Tribocco, Simone Frara, Angelo Armandi, Marco Astegiano, Giorgio Maria Saracco, Luciano Bertolusso, Davide Giuseppe Ribaldone

**Affiliations:** 1 Department of Medical Sciences, University of Turin, 10126 Turin, Italy; 2 A.S.L. CN2 Alba-Bra, 12051 Alba (CN), Italy; 3 Unit of Gastroenterology and Digestive Endoscopy, Michele e Pietro Ferrero Hospital, 12060 Verduno, Italy; 4 Unit of Gastroenterology, A.O.U. Città della Salute e della Scienza di Torino—Molinette Hospital, 10126 Turin, Italy

**Keywords:** burden, incidence, Crohn’s disease, prevalence, ulcerative colitis

## Abstract

The burden of inflammatory bowel diseases (IBD), including Crohn’s disease (CD) and ulcerative colitis (UC), is increasing worldwide. The aim of the present study was to investigate the clinical characteristics and the changing in epidemiology of IBD in the Healthcare District Bra, an area of North-West Italy accounting for 57,615 inhabitants as of 31 December 2021. Clinical and demographic data were retrieved from administrative databases and the medical records of general practitioners (*n* = 39) at Verduno Hospital. Prevalence and incidence rates were calculated for the time span 2016–2021 and compared to the 2001–2006 period. IBD prevalence was 321.2 per 100,000 population in 2021 and, compared with 2006 (200 per 100,000 population), the prevalence has increased at a rate of +46%. Similarly, the average incidence has increased from the period 2001–2006 (6.7 per 100,000 population/year) to the period 2016–2021 (18.0 per 100,000 population/year) at a rate of +169%; such an increase was greater for CD than UC. In the 2016–2021 period, the mean age at diagnosis was 42.0 ± 17.4 years and 30.9% required at least one hospitalization, while 10.9% of patients underwent at least one surgery. In conclusion, the prevalence and incidence of IBD distinctly increased over a two decade period in the Healthcare District Bra paralleling the results of previous surveys from other Italian regions. These data warrant specific interventions to improve patients’ management and resources’ allocation.

## 1. Introduction

Inflammatory bowel diseases (IBD) are chronic inflammatory conditions with a multifactorial etiopathogenesis that affect the gastrointestinal system. The two main entities are Crohn’s disease (CD) and ulcerative colitis (UC) [1]. The epidemiology of IBD is rapidly changing worldwide; the estimated prevalence (>0.3%) continues to rise in Western countries, with a high burden of IBD in North America, Oceania, and Europe [2]. The prevalence is also growing in newly industrialized countries in Africa, Asia, and South America mirroring the growing incidence of IBD observed in the 1990s in Western countries occurring with urbanization and the rapid socioeconomic development [3]. 

In Italy, the Global Burden of Diseases (GBD) estimated an IBD prevalence of 80.9 per 100,000 population in 1990 (56,469 cases) that increased to 93.8 per 100,000 population in 2017 (76,581 cases) (age-standardized percentage change: 16.0%), with a death rate over the study period increasing from 0.4 per 100,000 population in 1990 to 0.8 per 100,000 population in 2017 (age-standardized percentage change: 99.0%) [4]. However, local studies published in the last 10 years reported a significantly higher order of magnitude of IBD cases in Italy. The prevalence of IBD ranges from 442 per 100,000 population in the district of Milan (North Italy) to 187 per 100,000 population in Sardinia [5,6], with a lower prevalence of CD compared with UC in the whole country [5,6,7,8,9]. Incidence rates of CD varies from 6.1 to 17.9 per 100,000 population/year for CD and from 12.2 to 15.3 per 100,000 population/year for UC [5,6,7,8,9].

Considering the clinical relapsing course and the high probability of complications [10,11,12], in spite of a marginal reduction of life expectancy [13], IBD leads to a substantial socio-economic burden on the health-care system, in terms of hospitalization and pharmacologic treatments, and on society in terms of working productivity. Therefore, a correct estimation of the current epidemiological scenario is crucial for planning health-care policy and resource allocation.

The aim of the present study was to evaluate the clinical characteristics and the change in the epidemiology of IBD in a Healthcare District in the North-West of Italy.

## 2. Materials and Methods

### 2.1. Study Design

Data for the present retrospective, observational study were retrieved from an administrative database (resident population) and medical records of general practitioners (all general practitioners who are in charge of the entire population residing in the district were involved, total number of general practitioners = 39) and hospital service (Michele and Pietro Ferrero Hospital–Verduno) of A.S.L. CN2 from the Healthcare District of Bra (Cuneo, North–West Italy) that includes the municipalities of Bra, Cherasco, Sommariva del Bosco, Sommariva Perno, La Morra, Verduno, Sanfrè, Ceresole d’Alba, S.Vittoria d’Alba, Pocapaglia, and Narzole. In particular, data on the resident population of the district were retrieved from the official site of the Piedmont Region “PISTA”, which reports socio-demographic and economic information, including data on the structure and movements of the resident population according to province, municipality, sex, and age, health data, foreign residents, and censuses of general population [14].

We collected data from subjects with age ≥ 14 years (general practitioners and not pediatricians of free choice were involved) with a diagnosis of IBD (CD, UC, or IBD unclassified (IBDU)) within the time span 2016–2021. Patients were classified as IBDU in case of endoscopic, radiographic, and histologic evidence of chronic inflammatory bowel disease confined to the colon, but without fulfillment of diagnostic criteria for UC and CD. In detail, the following data (from IBD diagnosis to last follow-up visit) were retrieved, and recorded in a specific database: demographic features, data concerning the diagnosis of the disease (year of diagnosis, type, and location [15]), family history, hospitalization, and surgery for IBD, occurrence of neoplasms (intestinal and extra-intestinal), current and previous IBD therapy, death, and cause of death. Patients included had unique identifiers that ensured that there were no duplicate patient entries within the study period; in case of data retrieved from both the hospital service and general practitioner, medical records were reviewed and used to double-check the general practitioner data. Epidemiologic data estimated in the time span 2016–2021 were compared to those estimated in the same district in the time span 2001–2006 (the method of involvement of all general practitioners in the district was the same in the 2 periods [16]). Patients who had not died in the meantime or who had not changed residence were also included in the 2016–2021 analysis. Incidence and prevalence were computed both annually and as averages for 2016–2021, while as an average only for 2001–2006.

### 2.2. Statistical Analysis

The characteristics of the study cohort were reported as mean and standard deviation (SD) for continuous, normally distributed variables, and as absolute numbers (*n*) and percentages (%) for categorical data. Continuous variables were compared between groups by Students’ *t* test, while categorical data were analyzed by chi-squared (χ^2^) test. Standardized prevalence and incidence were reported as rates per 100,000 population. For all the analyses, we considered significant a *p* value < 0.05. Statistics were performed by MedCalc software v.20.104 (MedCalc Software Ltd., Ostend, Belgium).

## 3. Results

On 31 December 2021, the population in the Healthcare District of Bra area was 57,615 inhabitants. The total number of patients affected by IBD was 186, of whom 103 were males (M) and 83 were females (F) (Table 1); 70 patients had a diagnosis of CD (35 M and 35 F); 81 had a diagnosis of UC (47 M and 34 F); and 24 were affected by IBDU (13 M and 11 F). Concerning the remaining 11 patients (eight M and three F), we were unable to retrieve any data since they underwent instrumental investigations at the Verduno Hospital but were followed-up for their disease by other centers outside the Health Care District of Bra. These patients were included in the calculation of IBD incidence and prevalence, but were excluded from CD, UC, and IBDU subgroup analysis.

**Figure 1:**
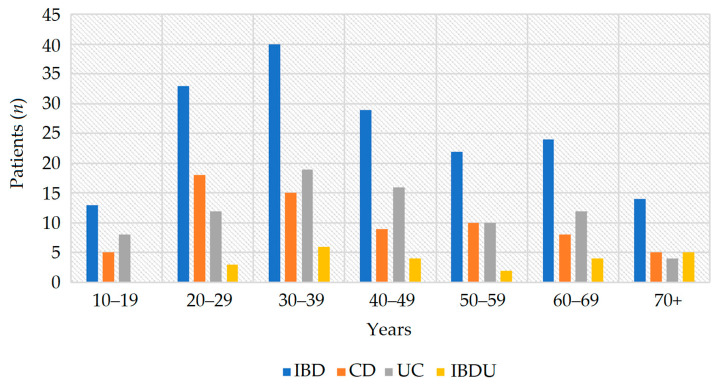
Distribution of IBD diagnosis according to age groups. Abbreviations: CD, Crohn’s disease; IBD, inflammatory bowel disease; IBDU, IBD unclassified; UC, ulcerative colitis.

The mean age at diagnosis was 42.0 ± 17.4 years for the whole IBD population (Figure 1). The mean age for CD, UC, and IBDU was 41.0 ± 17.2 years, 42.0 ± 16.8 years, and 50.0 ± 19.0 years, respectively. The M-to-F ratio was 1.2:1 for IBD, 1:1 for CD, 1.4:1 for UC, and 1.2:1 for IBDU. Immigrants born abroad comprised 20 out of 175 (11.4%) patients with IBD, 10 from East–Europe (Romania, Russia, and Balkan Countries) and 10 from Africa. Patients’ mean follow-up was 12.5 ± 10.3 years. No deaths occurred within the study period.

### 3.1. Clinical Characteristics

The diagnosis of IBD was mainly achieved by endoscopy (colonoscopy, video capsule, esophagogastroduodenoscopy) associated to a histological examination of bioptic samples (167 out of 175; 95.4%). In seven (4.0%) patients, the final diagnosis was achieved after a surgical procedure, while in one (0.6%) patient, IBD was diagnosed by imaging alone.

Among UC patients (*n* = 81), 16 (19.8%) had ulcerative proctitis (E1), five (6.2%) had left–side colitis (E2), and 60 (74.1%) had extensive (E3). Among CD patients (*n* = 70), the disease was confined to the ileum in 15 (21.4%) patients (L1), to the colon in 22 (31.4%) patients (L2), while 33 (47.1%) patients had an ileocolonic location (L3). Finally, in IBDU patients, the main localization of disease was in the colon alone (*n* = 7; 29.2%), followed by colon and rectum (*n* = 7; 29.2%), rectum alone (*n* = 5; 20.8 %), ileum alone (*n* = 3; 12.5%), ileum and colon (*n* = 1; 4.2%), and rectum and anus (*n* = 1; 4.2%).

Overall, 54 out of 175 (30.9%) IBD patients required at least one hospitalization; patients with CD required hospitalization more frequently than those with UC (30 (42.9%) vs. 17 (21.0%); *p* = 0.004). Regarding surgical procedures, 19 (10.9%) IBD patients underwent at least one surgery; patients with CD underwent surgery more frequently than those with UC (17 (24.3%) vs. 2 (2.9%); *p* < 0.001). In the whole IBD population, 11 out of 19 (57.9%) patients underwent surgery within 5 years from diagnosis.

Neoplasm occurred in 14 (8.0%) patients; six patients developed an intestinal neoplasm (all adenomas with mild dysplasia), while eight patients developed a miscellanea of extra-intestinal neoplasm, including pancreatic, uterine, upper lip carcinoma, laryngeal, skin, bladder, prostate, and breast cancer. No difference was observed in neoplasm incidence according to IBD type.

During the analyzed period, azathioprine or methotrexate was prescribed in 29 (16.6%) IBD patients (13 out of 70 (18.6%) CD patients, 14 out of 81 (17.3%) UC patients, two out of 24 (8.3%) IBDU patients). Biologic therapy and/or small molecule drugs were prescribed at least once in 34 out of 175 (19.4%) IBD patients (19 out of 70 (27.1%) CD patients and 15 out of 81 (18.5%) UC patients). Among the overall number of biologic therapy and/or small molecule prescriptions (*n* = 49), adalimumab accounted for the 53.1% (*n* = 26) of total prescriptions, followed by infliximab (*n* = 10; 20.4%), vedolizumab (*n* = 6; 12.2%), ustekinumab (*n* = 3; 6.1%), and tofacitinib (*n* = 3; 6.1%).

### 3.2. Prevalence

IBD prevalence in the Healthcare District of Bra on 31 December 2021 was 321.2 per 100,000 population. The municipality with the lowest prevalence was Ceresole d’Alba (167.5 per 100,000 population), while the municipality with the highest prevalence was Sommariva Perno (753.8 per 100,000 population). Compared with 2006 (146 IBD diagnosis) [16], IBD prevalence grew by 46% (*p* = 0.001) in 2021 (220 per 100,000 population vs. 321.2 per 100,000 population). The municipality with the greatest increase was Pocapaglia (+351%), followed by Sommariva Perno (+202%), and La Morra (+200%). Conversely, in the municipalities of Ceresole d’Alba and Sanfrè, we observed a decrease in IBD prevalence as there was a percentage decrease of −42% and −2%, respectively (Table 2). As per IBD type, CD prevalence increased significantly by 76% (*p* = 0.003), while no significant variation was observed for UC (+15%; *p* = 0.352) and IBDU (+39%; *p* = 0.352).

CD showed a lower prevalence than UC (121.5 per 100,000 vs. 138.9 per 100,000). The lowest CD prevalence was recorded in Verduno (no CD cases), while the highest was in Sommariva Perno (335.0 per 100,000 population). The lowest UC prevalence was recorded in La Morra (42.0 per 100,000 population), while the highest was in Sommariva Perno (376.9 per 100,000 population). Most of the municipalities recorded a higher prevalence for UC compared with CD, except for Ceresole d’Alba, Cherasco, La Morra, and Pocapaglia. IBDU showed a prevalence of 41.7 per 100,000 inhabitants, ranging from a minimum of 0 in Sanfrè and Ceresole D’Alba to a maximum of 203.3 per 100,000 population in Verduno.

### 3.3. Incidence

**Figure 2:**
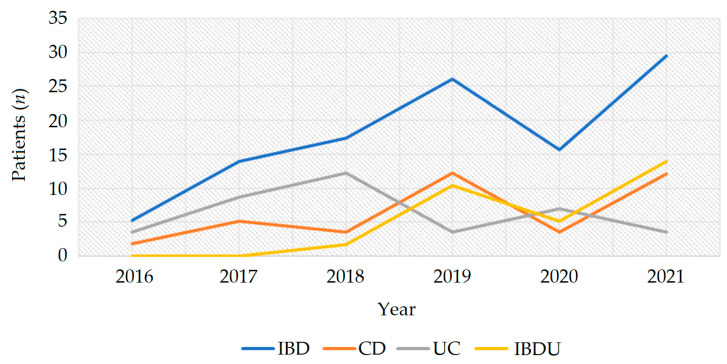
IBD incidence in the Healthcare District of Bra in the time span 2016–2021. Abbreviations: CD, Crohn’s disease; IBD, inflammatory bowel disease; IBDU, IBD unclassified; UC, ulcerative colitis.

The average IBD incidence rate in the Healthcare District of Bra in the time span 2016–2021 was 18.0 per 100,000 population/year. The average IBD incidence increase from 2001–2006 to 2016–2021 was 169%. The incidence rate estimated in the time span 2016–2021 was similar among CD and UC, while in the time-span 2001–2006 was higher for UC, with a percentage increase of 121% in CD and 73% in UC (Table 3 and Figure 2).

## 4. Discussion

The prevalence of IBDs has undergone an important phase of growth during the 21st century. This increase entails new organizational needs for the Healthcare System in order to improve patients’ management and to allow costs’ reduction. In the present study, we investigated the clinical features and the epidemiologic scenario of IBD in a Healthcare District of North-West Italy. As the main findings, we observed that IBD prevalence in the Healthcare District of Bra was 321.2 per 100,000 population in 2021, compared with 2006 (200 per 100,000 population), the prevalence has increased at a rate of +46%. Similarly, the average incidence has increased from the period 2001–2006 (6.7 per 100,000 population/year) to the period 2016–2021 (18.0 per 100,000 population/year) at a rate of +169%; such an increase was greater for CD than UC.

Despite some degree of variability, our data are in agreement with the results of previous epidemiologic studies carried out in Italy [5,6,7,8,9]. In particular, a previous Italian epidemiologic study carried out in the Barletta-Andria-Trani Province of the Apulia region, found an IBD prevalence of 187.2/100,000 (95% CI: 160.6–217.0) and an incidence of 16.2/100,000 (95% CI 12.5–20.7) per year [17]; taking into account that these data were published in 2013, they are consistent with our findings of an increase in IBD prevalence and incidence. Concerning CD and UC, we observed a prevalence of 121.5 per 100,000 population and 138.9 per 100,000 population, respectively. These data are lower than those reported from Northern Europe (CD: 143.0 per 100,000 population and UC: 297.9 per 100,000 population), but higher than those estimated in Asia (CD: 27.1 per 100,000 population and UC: 55.4 per 100,000 population) and Africa (CD: 19.0 per 100,000 population and UC: 10.6 per 100,000 population) [2]. Conversely, the incidence of CD observed in our study was similar to that reported in Northern Europe (6.4 per 100,000 population/year vs. 5.7 per 100,000 population/year, respectively), while the incidence of UC was distinctly lower (6.4 per 100,000 population/year vs. 29.8 per 100,000 population/year, respectively). As compared to data from Asia (CD: 4.2 per 100,000 population/year and UC: 3.3 per 100,000 population/year) and Africa (CD: 5.9 per 100,000 population/year and UC: 3.3 per 100,000 population/year), the incidence of both CD and UC was higher in our population [2]. There was a decline of new diagnoses of IBD in 2020, probably caused by a delay in the first visits due to the concomitant presence of the SARS-CoV-2 pandemic; these diagnoses, which in the absence of a pandemic would have occurred in 2020, presumably were performed in 2021, creating an overestimation in that year. This is confirmed by an international survey, which found that 85% of endoscopy units have reduced the volume of procedures by more than 50% due to the pandemic [18].

From a clinical point of view, the average age at diagnosis was about 40 years which, having also excluded patients <14 years of age, confirms that IBD arises at a young age and remains a condition for the patient’s entire life [19]. The rate of hospitalizations, but above all that of surgical interventions, although confirmed to be higher for CD (about 25%) than for UC (about 3%), is lower than that reported in the literature (about 60% in CD, about 10% in UC) [20]. This probably derives from the fact that our study was not based on a population of a tertiary care center but collected data from the entire population affected by IBD living in a given territory, and therefore more closely reflects the disease trend in the general IBD population. With regard to the use of advanced therapies (biological drugs and small molecules), a greater use is confirmed in CD (about 30%) compared with UC (about 20%); moreover, the percentage of the use of advanced therapies does not differ from the literature data (more than 30% in CD, more than 10% in UC) [21]. Our figure would perhaps have been even higher if we had also included the pediatric population [22].

The main strengths of this study are represented by involving all the general practitioners operating in the district in which we analyzed the prevalence and incidence of IBD and having checked these data with those of the only hospital in the district. The main limitations consist in the fact that the data on the characteristics of the IBD were collected by the medical records of the general practitioners of the patients and that the second check with the data present in the medical records of the district hospital could only be done for the patients by a specialist of that hospital.

## 5. Conclusions

In conclusion, the prevalence of IBD as of 31 December 2021 in the Health Care District of Bra was 321.2 per 100,000 inhabitants, with a significant increase of 46% compared with 2006 (220 per 100,000 inhabitants); the average incidence of IBD in the period 2016–2021 was 18 per 100,000 inhabitants/year, with an increase of 169% compared with the period 2001–2006 (6.7 per 100,000 inhabitants/year) and the increase was greater for CD than for UC. The incidence and prevalence values that emerged from our study were lower than the values that emerged from other studies conducted in Italy, but comparable to the values estimated for Europe.

These data are essential for understanding the epidemiological trend and for planning health resources for these patients.

## Figures and Tables

**Table 1:** Patients affected by IBD in the Healthcare District of Bra.

Municipality	Population (*n*)	IBD (*n*)	CD (*n*)	UC (*n*)	IBDU (*n*)	IBD Witout Clinical Data (*n*)
Bra	25,996	87 M = 48; F = 39	31 M = 14; F = 17	39 M = 24; F = 15	12 M = 7; F = 5	5 M = 3; F = 2
Ceresole d’Alba	1791	3 M =1; F = 2	2 M = 0; F = 2	1 M = 1; F = 0	0	0
Cherasco	8098	18 M = 9; F = 9	11 M = 6; F = 5	4 M = 1; F = 3	3 M = 2; F = 1	0
Santa Vittoria d’Alba	2504	11 M = 7; F = 4	3 M = 3; F = 0	6 M = 3; F = 3	2 M = 1; F = 1	0
La Morra	2379	5 M = 3; F = 2	2 M = 1; F = 1	1 M = 1; F = 0	1 M = 0; F = 1	1 M = 1; F = 0
Narzole	3014	6 M = 5; F = 1	1 M = 1; F = 0	4 M = 3; F = 1	1 M = 1; F = 0	0
Pocapaglia	2848	9 M = 5; F = 4	4 M = 3; F = 1	2 M = 1; F = 1	2 M = 0; F = 2	1 M = 1; F = 0
Sanfrè	2632	9 M = 5; F = 4	2 M = 1; F = 1	6 M = 4; F = 2	0	1 M = 0, F = 1
Sommariva Del Bosco	5473	18 M = 11; F = 7	6 M = 3; F = 3	8 M = 5; F = 3	1 M = 0; F = 1	3 M = 3; F = 0
Sommariva Perno	2388	18 M = 8; F = 10	8 M = 3; F = 5	9 M = 4; F = 5	1 M = 1; F = 0	0
Verduno	492	2 M = 1; F = 1	0	1 M = 0; F = 1	1 M = 1; F = 0	0
Healthcare Disctric of Bra	57,615	186 M = 103; F = 83	70 M = 35; F = 35	81 M = 47; F = 34	24 M = 13; F = 11	11 M = 8; F = 3

Population with age ≥14 years on 31 December 2021. Abbreviations: CD, Crohn’s disease; F, female; IBD, inflammatory bowel disease; IBDU, IBD unclassified; M, male; *n*, number; UC, ulcerative colitis.

**Table 2:** IBD prevalence in the Healthcare District of Bra according to time-period.

Municipality	IBD Prevalence (2021)	IBD Prevalence (2006)	Variation (%) 2006–2021	CD Prevalence (2021)	UC Prevalence (2021)	IBDU Prevalence (2021)
Bra	334.4	250	+34%	119.2	150.0	46.0
Ceresole d’Alba	167.5	290	−42%	111.7	55.8	0
Cherasco	222.2	160	+39%	135.8	49.4	37.0
Santa Vittoria d’Alba	439.3	270	+63%	119.8	239.6	79.9
La Morra	210.2	70	+200%	84.2	42.0	42.0
Narzole	199.1	150	+33%	33.2	132.7	33.2
Pocapaglia	315.9	70	+351%	140.4	70.2	70.2
Sanfrè	342.0	350	−2%	76.0	228.0	0
Sommariva Del Bosco	310.7	250	+24%	109.6	128.0	18.3
Sommariva Perno	753.8	250	+202%	335.0	376.9	41.9
Verduno	406.6	380	+7%	0	203.3	203.3
Health Care Disctric of Bra	321.2	220	+46%	121.5	138.9	41.7

Prevalence is reported per 100,000 population. Abbreviations: CD, Crohn’s disease; IBD, inflammatory bowel disease; IBDU, IBD unclassified; UC, ulcerative colitis.

**Table 3:** IBD incidence in the Healthcare District of Bra according to time-period.

	2016	2017	2018	2019	2020	2021	2016–2021 (Mean ± SD)	2001–2006 (Mean)	Variation (%) 2006–2021
IBD	5.3	13.9	17.4	26.1	15.7	29.5	18.0 ± 8.0	6.7	169%
CD	1.8	5.2	3.5	12.2	3.5	12.1	6.4 ± 4.2	2.9	121%
UC	3.5	8.7	12.2	3.5	7.0	3.5	6.4 ± 3.3	3.7	73%
IBDU	0	0	1.7	10.4	5.2	13.9	5.2 ± 5.3	N/A	

Incidence is reported per 100,000 population/year. Abbreviations: CD, Crohn’s disease; IBD, inflammatory bowel disease; IBDU, IBD unclassified; N/A, not available; SD, standard deviation; UC, ulcerative colitis.

## Data Availability

The data presented in this study are available upon request from the corresponding author.
